# The effect of different depths of medial heel skive on plantar pressures

**DOI:** 10.1186/1757-1146-5-20

**Published:** 2012-08-13

**Authors:** Daniel R Bonanno, Cheryl Y Zhang, Rose C Farrugia, Matthew G Bull, Anita M Raspovic, Adam R Bird, Karl B Landorf

**Affiliations:** 1 Department of Podiatry, Faculty of Health Sciences, La Trobe University, Melbourne, Vic, 3086, Australia; 2 Musculoskeletal Research Centre, Faculty of Health Sciences, La Trobe University, Melbourne, Vic, 3086, Australia

**Keywords:** Foot orthoses, Medial heel skive, Foot pronation, Flat-feet, Plantar pressures

## Abstract

**Background:**

Foot orthoses are often used to treat lower limb injuries associated with excessive pronation. There are many orthotic modifications available for this purpose, with one being the medial heel skive. However, empirical evidence for the mechanical effects of the medial heel skive modification is limited. This study aimed to evaluate the effect that different depths of medial heel skive have on plantar pressures.

**Methods:**

Thirty healthy adults (mean age 24 years, range 18–46) with a flat-arched or pronated foot posture and no current foot pain or deformity participated in this study. Using the in-shoe pedar-X® system, plantar pressure data were collected for the rearfoot, midfoot and forefoot while participants walked along an 8 metre walkway wearing a standardised shoe. Experimental conditions included a customised foot orthosis with the following 4 orthotic modifications: (i) no medial heel skive, (ii) a 2 mm medial heel skive, (iii) a 4 mm medial heel skive and (iv) a 6 mm medial heel skive.

**Results:**

Compared to the foot orthosis with no medial heel skive, statistically significant increases in peak pressure were observed at the medial rearfoot – there was a 15% increase (p = 0.001) with the 4 mm skive and a 29% increase (p < 0.001) with the 6 mm skive. No significant change was observed with the 2 mm medial heel skive. With respect to the midfoot and forefoot, there were no significant differences between the orthoses.

**Conclusions:**

This study found that a medial heel skive of 4 mm or 6 mm increases peak pressure under the medial rearfoot in asymptomatic adults with a flat-arched or pronated foot posture. Plantar pressures at the midfoot and forefoot were not altered by a medial heel skive of 2, 4 or 6 mm. These findings provide some evidence for the effects of the medial heel skive orthotic modification.

## Background

Foot orthoses are commonly used to treat a wide range of musculoskeletal pathologies [1]. In particular, foot orthoses are frequently used for conditions associated with foot pronation, such as patello-femoral pain syndrome [2]. The mechanism of action of foot orthoses is still not clear, however there is evidence that they provide small but significant changes to the mechanical function of the lower limb [3]. Specifically, foot orthoses are thought to provide beneficial outcomes by altering kinematics, kinetics, and muscle activity [3-6].

There are many types of orthotic styles, materials and modifications that are designed to enhance the effects of foot orthoses [7]. One such modification, the *medial heel skive*, is a technique that was developed with the intention of improving the ability of a foot orthosis to control excessive foot pronation [8]. The medial heel skive technique creates a varus wedge within the heel cup of a foot orthosis [8]. This wedge is intended to increase the force acting on the medial plantar heel, which is hypothesised to increase the supination moment acting across the subtalar joint axis [8]. Different depths of medial heel skive can be prescribed, with greater depths indicated when greater pronatory control is desired [8].

Despite its use clinically, empirical evidence for the mechanical effects of the medial heel skive modification is lacking. As such, a better understanding of how it affects the foot biomechanically will help guide its use. Therefore, this study aimed to evaluate the effect that different depths of medial heel skive have on plantar pressures in adults with a flat-arched or pronated foot posture.

## Methods

### Participants

Thirty adult participants with a flat-arched or pronated foot posture were recruited between July and September 2010 via advertisements at a local university. Participants were eligible for inclusion if they were aged 18 years or older and were classified as having a flat-arched or pronated foot posture according to one of two clinical techniques, the normalised navicular height truncated measure (NNHT) [9] and the six-item Foot Posture Index (FPI-6) [10]. The NNHT and FPI-6 are both reliable and valid tools used to determine static foot posture [9,11]. The NNHT is the ratio of navicular height relative to the truncated foot length – with a lower ratio indicative of a flatter-arched foot [9]. The FPI-6 uses six criterion-based observations, which are each scored on a 5-point scale (range −2 to +2); these are then summated to produce a final score which can range from −12 (very supinated) to +12 (very pronated) [11]. Participants were determined to have a flat-arched or pronated foot posture if their static foot posture was greater or equal to one standard deviation from the population mean, as determined in normative studies elsewhere, in the direction of a flatter or more pronated foot, for either the NNHT (<0.24) [9] or FPI-6 (> + 7) [10]. Participants were excluded from the study if they had foot or leg pain, a history of foot surgery or were unable to speak English. The study was approved by the institutional ethics committee (application number FHEC10/57) and written informed consent was obtained from all participants. The characteristics of the participants are shown in Table 1.

**Table 1:** Participant characteristics (N = 30)

**Characteristic**	**Mean**	**Standard deviation**	**Range**
Age (years)	24.1	6.4	18 to 46
Height (m)	1.73	0.10	1.50 to 1.92
Weight (kg)	71.5	13.9	50.8 to 102.1
Body mass index (kg/m2)	23.8	3.2	18.8 to 32.6
FPI-6	7.0	2.0	3.0 to 10.0
NNHT	0.20	0.03	0.14 to 0.23

### Interventions

All foot orthoses, footwear and sockettes used in the study were commercially available at the time of testing. The canvas athletic footwear (Dunlop Volley, Pacific Dunlop Ltd, Melbourne, Australia) and sockettes, a thin stocking-like foot cover with no plantar seams, were standardised to minimise their influence on plantar pressures across participants.

**Figure 1:**
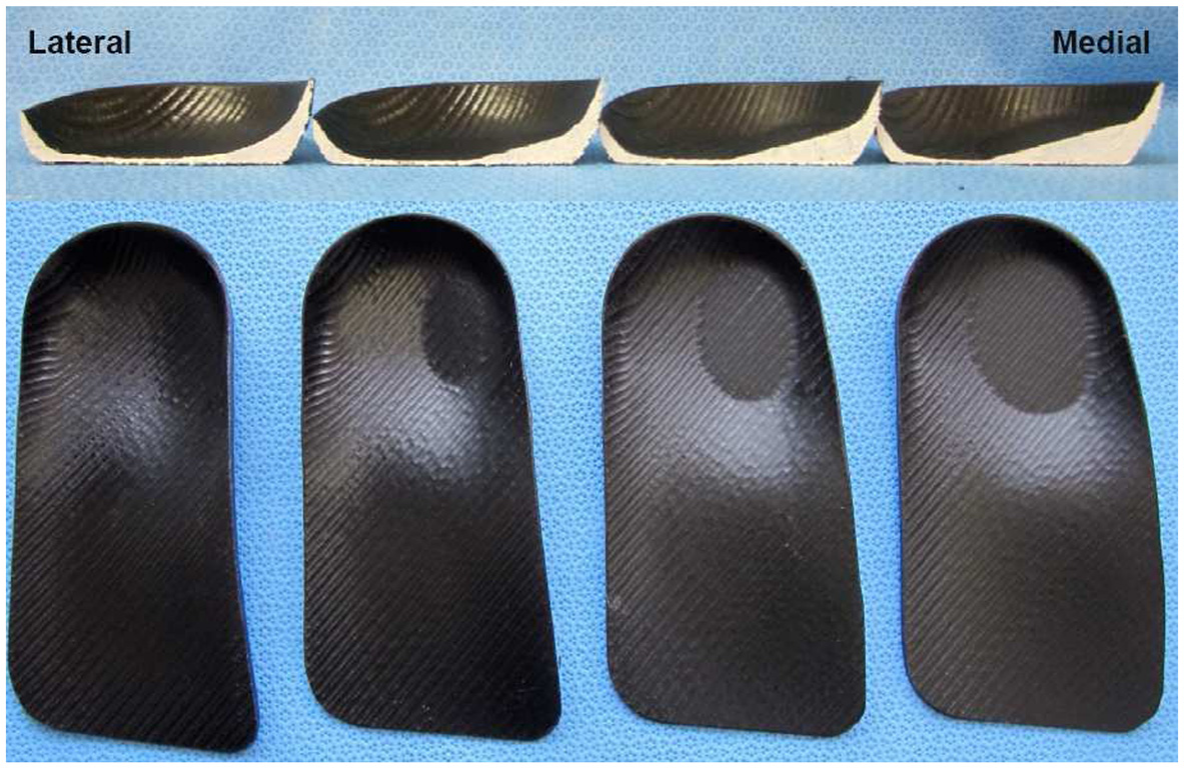
**Cross-sectional (top) and superior (bottom) view of the four experimental conditions.** Left to right: (i) orthosis with no medial heel skive; (ii) orthosis with a 2 mm medial heel skive; orthosis with a 4 mm medial heel skive; and (iv) orthosis with a 6 mm medial heel skive.

The 4 orthotic conditions analysed were (Figure 1):

(i)  Orthosis with no medial heel skive (control condition),

(ii)  Orthosis with a 2 mm medial heel skive,

(iii)  Orthosis with a 4 mm medial heel skive,

(iv)  Orthosis with a 6 mm medial heel skive.

**Figure 2:**
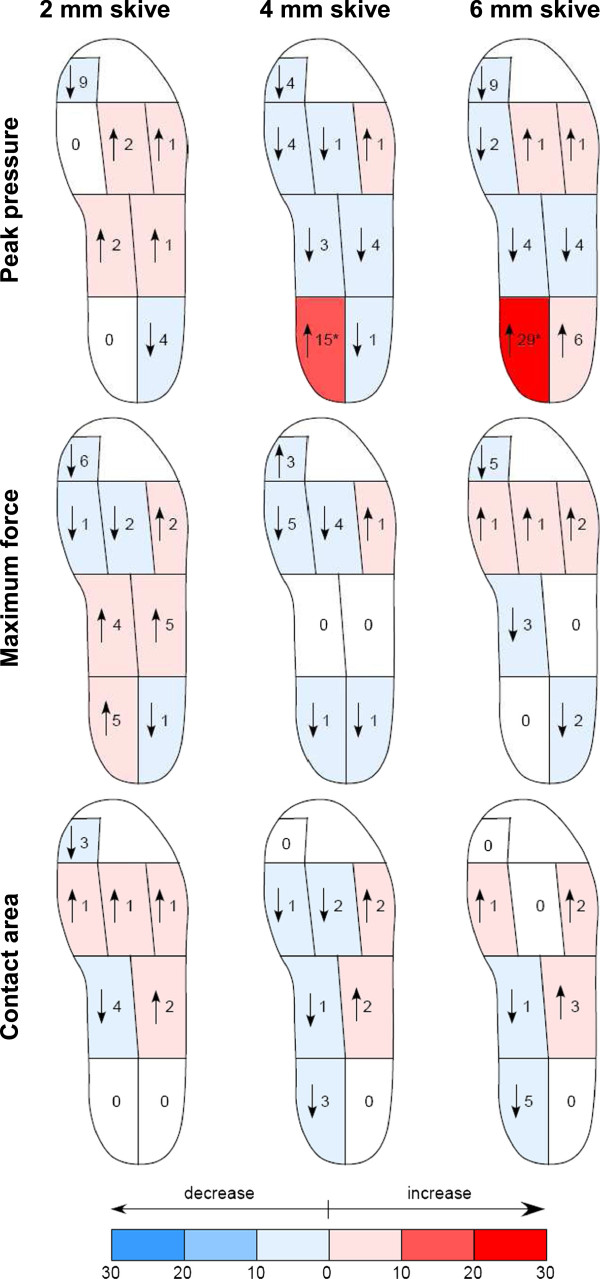
**Percentage change of peak pressure, maximum force and contact area for the mask areas of each foot orthotic condition with a medial heel skive compared to the foot orthosis with no medial heel skive (N=30).** Significant (p<0.05) changes marked with an asterisk (*).

Plaster cast impressions were taken of each participant’s feet using the suspension technique [12]. The foot orthoses used in this study represented the typical prescription habits of Australian and New Zealand podiatrists [7]. The orthoses were a modified Root style device balanced to the neutral calcaneal stance position and made with a polypropylene shell. The shell thickness was either 4.0 mm or 4.5 mm, dependent on the participant’s body weight. Polypropylene of 4.0 mm was used for participants with a body mass of less than 75 kg and 4.5 mm for participants with a body mass of equal to or greater than 75 kg [13]. Orthoses were manufactured by a commercial laboratory (Virtual Orthotics Pty Ltd, Sydney, Australia) using a computer-aided design and a computer-aided manufacturing (CAD–CAM) process, whereby each orthosis was directly milled from a polypropylene block. As CAD–CAM procedure ensures consistency in the design and manufacturing of the orthoses the only variation between the devices was the depth of a medial heel skive modification [14] (Figure 2).

Typically, the medial heel skive modification is created by removing a portion of the plantar medial heel of the positive foot mould, also known as the positive cast [8]. The heel of the positive cast is initially divided into transverse thirds and a longitudinal cut (commonly 2, 4 or 6 mm deep) is made into the cast where the medial and middle third of the heel meet [8]. The medial aspect of the plantar heel is removed on a 15 degree angle until the marked depth has been reached [8]. As a result of the modification, the resultant orthosis has a varus wedge within the heel cup [8]. A deeper medial heel skive results in a more prominent varus wedge that covers a greater area under the heel (Figure 1).

### Apparatus

Plantar pressures were measured using the pedar-X® in-shoe system (Novel GmbH, Munich, Germany), which has been shown to exhibit a high level of accuracy, repeatability and validity [15-17]. Each pedar® insole comprises of 99 capacitive sensors embedded in a 2 mm thick insole. The pedar® insoles were calibrated with the trublu® calibration device prior to the commencement of the study (Novel GmbH, Munich, Germany). Plantar pressures were recorded in accordance with the manufacturer’s guidelines at a frequency of 50 Hz.

### Procedures

The foot orthoses were issued two weeks prior to data collection to allow participants to acclimatise to them prior to testing. During this time, participants wore their everyday footwear. Participants were required to document the wear time of each orthotic condition to ensure that all of the orthoses were worn for an equal amount of time.

Following the familiarisation period, participants presented to the La Trobe University Health Science Clinic (Melbourne, Australia) for data collection. Participants were issued with standardised sockettes and footwear. Appropriately sized pedar® insoles were placed between the foot and the orthotic condition to be tested. All participants were instructed to walk at their normal comfortable speed along an eight metre walkway. If a walking trial was not completed within 5% of the original walking time it was eliminated and repeated to ensure walking speed did not affect plantar pressures [18]. Each participant undertook 4 walking trials for each of the 4 orthotic conditions. To minimise the effects of acceleration and deceleration steps, only the middle 4 steps were used for data analysis. The 16 steps (4 steps from 4 trials) were averaged for each of the 4 orthotic conditions.

The 4 orthotic conditions were tested in random order to minimise potential sequencing effects. Participants were blinded as to which depth of medial heel skive was being tested. Investigators were not blinded due to the difficulty in concealing each orthotic condition.

### Outcome measures

The primary outcome measures were peak pressure, maximum force and contact area under the medial and lateral rearfoot. Secondary outcomes measures included contact time under the whole foot and peak pressure, maximum force and contact area under the medial and lateral midfoot, hallux, and medial, central and lateral forefoot.

### Statistical analysis

A specific sample size calculation was not performed prior to the study due to the uncertainty of what constitutes a clinically worthwhile difference (i.e. a minimal important difference) for the effects of the medial heel skive modification on rearfoot plantar pressures. Instead, we based our sample size on the decision to use parametric statistics; that is, a sample size of 30 is generally considered appropriate, providing the data is normally distributed, to be able to use parametric statistical analysis [19]. In addition, significant differences in the variables being investigated in this study have been detected in previous orthotic studies with similar or smaller samples [20-22].

The plantar pressure data were entered into the pedar® analysis program. Percentage sized masks were applied to the rearfoot (proximal 31% of foot length), midfoot (middle 19% of foot length) and forefoot (distal 50% of foot length) [23]. The rearfoot and midfoot masks were subsequently bisected into medial and lateral halves. The forefoot mask consisted of four regions: the hallux, medial forefoot (1^st^ metatarsophalangeal region), central forefoot (2^nd^ and 3^rd^ metatarsophalangeal region) and lateral forefoot (4^th^ and 5^th^ metatarsophalangeal region). Lateral digits were excluded from data analysis due to previously reported low yield and high variability for plantar pressure data [20].

All statistical analysis was performed using the computer program Statistical Package for the Social Sciences (SPSS) Version 17.0 (SPSS Inc, Chicago, Illinois). Data were explored for normality prior to inferential analysis – data that were not normally distributed were transformed prior to inferential analysis. In this project, all variables identified as not normally distributed required 'reflect and square root' transformation. A one-way repeated measures analysis of variance (ANOVA) with Bonferroni-adjusted post-hoc test was used to compare means between each of the orthotic conditions. Differences between orthotic conditions were considered statistically significant if *p* < 0.05.

## Results

The sample of 30 participants was made up of 18 females (60%) and 12 males (40%). A summary of participant characteristics and foot anthropometric data is provided in Table 1.

Several statistically significant differences in peak pressure, maximum force and contact area were found between the 4 orthotic conditions in the rearfoot (Figure 2). In contrast, no significant plantar pressure differences were found between the devices in the midfoot and forefoot. As contact time did not differ across the four orthotic conditions it can be assumed that any differences in plantar pressures can be attributed to the conditions being analysed and not a variation in walking speed (Table 2).

**Table 2:** Comparison of the mean (SD) contact time for each of the conditions (N=30)

		**Contact time (ms)**	
**Condition**	**Mean (SD)**	**% change**	***p*-value**
Orthosis with no heel skive	677.7 (78.3)	n/a	n/a
Orthosis with 2 mm heel skive	677.4 (73.7)	0%	1.000
Orthosis with 4 mm heel skive	681.4 (71.3)	+1%	1.000
Orthosis with 6 mm heel skive	675.7 (73.5)	0%	1.000

Note: % change is relative to the orthosis with no heel skive.

### Medial rearfoot

Compared to no medial heel skive, significant increases in peak pressure were observed at the medial rearfoot (Table 3). There was a 15% increase (p = 0.001) and a 29% increase (p < 0.001) with the 4 mm and 6 mm medial heel skive respectively. In contrast, the 2 mm skive provided no significant change in peak pressure (p > 0.05). There were also significant differences between the various depths of skive. The 4 mm (p < 0.001) and 6 mm (p < 0.001) skives produced significant increases in peak pressure when compared to the 2 mm skive. Similarly, the 6 mm skive produced a significant increase in peak pressure compared to the 4 mm skive (p < 0.001).

**Table 3:** Mean values (SD) for the medial and lateral rearfoot (N=30)

				**Medial rearfoot**					
	**Peak pressure (kPa)**			**Maximum force (%BW)**			**Contact area (cm**^2^**)**		
**Condition**	**Mean (SD)**	**% change**	***p*-value**	**Mean (SD)**	**% change**	***p*-value**	**Mean (SD)**	**% change**	***p*-value**
Orthosis with no heel skive	205.7 (38.1)	n/a	n/a	28.0 (8.8)	n/a	n/a	20.3 (2.3)	n/a	n/a
Orthosis with 2 mm heel skive	205.2 (43.2)	0%	1.00	29.3 (10.1)	5%	1.000	20.4 (3.0)	0%	1.000
Orthosis with 4 mm heel skive	237.0 (52.5)	+15%	0.001^*#^	27.7 (8.8)	-1%	1.000	19.7 (2.6)	-3%	0.839
Orthosis with 6 mm heel skive	265.2 (51.1)	+29%	<0.001^*#†^	28.0 (10.8)	0%	1.000	19.3 (2.8)	-5%	0.054^#^
				**Lateral rearfoot**					
	**Peak pressure (kPa)**			**Maximum force (%BW)**			**Contact area (cm**^2^**)**		
**Condition**	**Mean (SD)**	**% change**	***p*-value**	**Mean (SD)**	**% change**	***p*-value**	**Mean (SD)**	**% change**	***p*-value**
Orthosis with no heel skive	247.3 (65.7)	n/a	n/a	46.8 (8.8)	n/a	n/a	23.0 (2.2)	n/a	n/a
Orthosis with 2 mm heel skive	236.2 (46.3)	-4%	0.751	46.2 (8.9)	-1%	1.000	23.0 (2.2)	0%	1.000
Orthosis with 4 mm heel skive	244.0 (61.0)	-1%	1.000	46.2 (9.0)	-1%	1.000	23.1 (2.1)	0%	0.623
Orthosis with 6 mm heel skive	263.3 (66.4)	+6%	1.000#†	46.0 (9.3)	-2%	1.000	23.1 (2.2)	0%	0.534

^*^ Mean difference significant at the 0.05 level (Bonferroni adjusted) compared to the unmodified orthosis.

^#^ Mean difference significant at the 0.05 level (Bonferroni adjusted) compared to the orthosis with a 2 mm heel skive.

^†^ Mean difference significant at the 0.05 level (Bonferroni adjusted) compared to the orthosis with a 4 mm heel skive.

There were no differences in maximum force among the orthotic conditions (p > 0.05) and only minor differences in contact area. When compared to no medial heel skive, none of the foot orthoses with a skive had a significant effect on contact area in the medial rearfoot. However, when the various depths of skive were compared the 6 mm skive significantly reduced contact area compared to the 2 mm skive (p < 0.004).

### Lateral rearfoot

Compared to no medial heel skive, none of the orthoses with a skive provided a statistically significant change in contact area, maximum force or peak pressure in the lateral rearfoot (Table 3). However, there were differences in peak pressure between the various depths of skive. The 6 mm heel skive produced a significant increase in peak pressure in the lateral rearfoot compared to the 2 mm (p < 0.016) and 4 mm (p < 0.048) heel skives.

### Midfoot and forefoot

There were no differences in peak pressure, maximum force, or contact area (p > 0.05) between the orthotic conditions at any of the midfoot (Table 4) or forefoot (Table 5) masks.

**Table 4:** Mean values (SD) for the medial and lateral midfoot (N=30)

				**Medial midfoot**					
	**Peak pressure (kPa)**			**Maximum force (%BW)**			**Contact area (cm**^2^**)**		
**Condition**	**Mean (SD)**	**% change**	***p*-value**	**Mean (SD)**	**% change**	***p*-value**	**Mean (SD)**	**% change**	***p*-value**
Orthosis with no heel skive	81.9 (27.2)	n/a	n/a	8.6 (3.8)	n/a	n/a	14.5 (3.6)	n/a	n/a
Orthosis with 2 mm heel skive	83.7 (23.1)	+2%	1.000	8.9 (4.4)	+4%	1.000	13.9 (4.3)	-4%	1.000
Orthosis with 4 mm heel skive	79.5 (24.1)	-3%	1.000	8.5 (4.3)	-0.4%	1.000	14.3 (3.8)	-1%	1.000
Orthosis with 6 mm heel skive	79.0 (25.7)	-4%	1.000	8.3 (3.9)	-3%	1.000	14.3 (3.4)	-1%	1.000
				**Lateral midfoot**					
	**Peak pressure (kPa)**			**Maximum force (%BW)**			**Contact area (cm**^2^**)**		
**Condition**	**Mean (SD)**	**% change**	***p*-value**	**Mean (SD)**	**% change**	***p*-value**	**Mean (SD)**	**% change**	***p*-value**
Orthosis with no heel skive	91.2 (29.1)	n/a	n/a	11.9 (4.5)	n/a	n/a	15.9 (2.9)	n/a	n/a
Orthosis with 2 mm heel skive	92.3 (26.9)	+1%	1.000	12.5 (4.6)	+5%	1.000	16.1 (2.7)	+2%	1.000
Orthosis with 4 mm heel skive	87.2 (23.8)	-4%	1.000	11.9 (4.3)	0	1.000	16.2 (2.7)	+2%	1.000
Orthosis with 6 mm heel skive	87.9 (26.7)	-4%	1.000	11.9 (4.3)	0	1.000	16.3 (2.7)	+3%	0.270

*Mean difference significant at the 0.05 level (Bonferroni adjusted) compared to the unmodified orthosis.

**Table 5:** Mean values (SD) for the medial, central and lateral forefoot and hallux (N=30).

				**Medial forefoot**					
	**Peak pressure (kPa)**			**Maximum force (%BW)**			**Contact area (cm**^2^**)**		
**Condition**	**Mean (SD)**	**% change**	***p*-value**	**Mean (SD)**	**% change**	***p*-value**	**Mean (SD)**	**% change**	***p*-value**
Orthosis with no heel skive	238.8 (75.9)	n/a	n/a	20.7 (7.0)	n/a	n/a	13.4 (2.0)	n/a	n/a
Orthosis with 2 mm heel skive	238.2 (68.8)	-0.3%	1.000	20.4 (6.4)	-1%	1.000	13.5 (1.9)	+1%	1.000
Orthosis with 4 mm heel skive	228.9 (75.6)	-4%	1.000	19.7 (6.0)	-5%	1.000	13.2 (2.1)	-1%	1.000
Orthosis with 6 mm heel skive	233.7 (69.1)	-2%	1.000	20.9 (5.9)	1%	1.000	13.5 (1.9)	+1%	1.000
				**Central forefoot**					
	**Peak pressure (kPa)**			**Maximum force (%BW)**			**Contact area (cm**^2^**)**		
**Condition**	**Mean (SD)**	**% change**	***p*-value**	**Mean (SD)**	**% change**	***p*-value**	**Mean (SD)**	**% change**	***p*-value**
Orthosis with no heel skive	295.1 (77.0)	n/a	n/a	34.2 (7.5)	n/a	n/a	16.5 (1.7)	n/a	n/a
Orthosis with 2 mm heel skive	301.8 (78.5)	2%	0.900	33.5 (7.9)	-2%	1.000	16.6 (1.6)	1%	1.000
Orthosis with 4 mm heel skive	292.4 (77.1)	-1%	1.000	32.9 (8.5)	-4%	1.000	16.2 (1.8)	-2%	1.000
Orthosis with 6 mm heel skive	302.3 (81.6)	1%	1.000	34.4 (7.0)	1%	1.000	16.5 (1.7)	0%	1.000
				**Lateral forefoot**					
	**Peak pressure (kPa)**			**Maximum force (%BW)**			**Contact area (cm**^2^**)**		
**Condition**	**Mean (SD)**	**% change**	***p*-value**	**Mean (SD)**	**% change**	***p*-value**	**Mean (SD)**	**% change**	***p*-value**
Orthosis with no heel skive	248.4 (56.6)	n/a	n/a	22.6 (4.8)	n/a	n/a	17.0 (1.7)	n/a	n/a
Orthosis with 2 mm heel skive	251.9 (63.5)	+1%	1.000	23.0 (5.2)	+2%	1.000	17.2 (1.6)	+1%	1.000
Orthosis with 4 mm heel skive	254.3 (61.9)	+1%	1.000	22.8 (5.2)	+1%	1.000	17.3 (1.7)	+2%	1.000
Orthosis with 6 mm heel skive	257.2 (63.6)	+1%	1.000	23.0 (5.0)	+2%	1.000	17.3 (1.7)	+2%	1.000
				**Hallux**					
	**Peak pressure (kPa)**			**Maximum force (%BW)**			**Contact area (cm**^2^**)**		
**Condition**	**Mean (SD)**	**% change**	***p*-value**	**Mean (SD)**	**% change**	***p*-value**	**Mean (SD)**	**% change**	***p*-value**
Orthosis with no heel skive	310.6 (93.9)	n/a	n/a	15.6 (6.9)	n/a	n/a	6.0 (0.9)	n/a	n/a
Orthosis with 2 mm heel skive	282.4 (79.9)	-9%	0.667	14.7 (6.7)	-6%	1.000	5.8 (1.1)	-3%	0.661
Orthosis with 4 mm heel skive	297.2 (96.8)	-4%	1.000	16.1 (6.6)	+3%	1.000	6.0 (1.1)	0%	1.000
Orthosis with 6 mm heel skive	284.0 (80.5)	-9%	0.499	14.9 (6.8)	-4%	1.000	6.0 (1.0)	0%	1.000

* Mean difference is significant at the 0.05 level (Bonferroni adjusted) compared to the unmodified orthosis.

## Discussion

The aim of this study was to evaluate the effect of differing depths of medial heel skive on plantar pressures. A medial heel skive incorporated into a foot orthosis has been hypothesised by Kirby [8] to increase and medially shift the force acting on the medial, plantar heel. It is thought that such an increase in force medially has a concomitant decrease in the force to the lateral, plantar heel. Therefore, the effect that the medial heel skive modification has on force applied to the rearfoot is of clinical importance as it may reduce excessive rearfoot pronation.

The findings of this study support that significant increases in peak pressure in the medial rearfoot can be achieved with a 4 mm (15%) and 6 mm (29%) medial heel skive in asymptomatic individuals with flat-arched or pronated feet. In contrast, a 2 mm skive had no significant effect on plantar pressures in the medial rearfoot in the same cohort. The effect of these changes on kinematic motion in the rearfoot is still unknown. Kirby has suggested that if the increase in force provided by an orthosis with a medial heel skive occurs more medial to the subtalar joint (rearfoot) axis it will increase the supination moment about the joint, which would assist in controlling excessive pronation [8]. Unfortunately, we did not find an increase in force with an increase in the depth of skive. Instead, as the depth of skive increased, peak pressure increased as a result of a decrease in contact area. However, this does not preclude a change in the centre of the resultant force from the orthosis (referred to by Kirby [8] as the ‘centre of the orthotic reactive force’) relative to the subtalar joint axis. If such a change did occur as a result of the change in contact area, this would lead to an increase in supination moment about the subtalar joint axis, and may result in a kinematic change, although we do not have data at this stage to support this premise.

The 6 mm medial heel skive was the only orthotic condition to increase (6%) lateral rearfoot peak pressure compared to the unmodified orthosis, although the increase was not statistically significant. However, because the 2 mm and 4 mm heel skives decreased lateral rearfoot peak pressure, the increase provided by the 6 mm heel skive was significantly greater than the 2 mm (p < 0.016) and 4 mm (p < 0.048) skives. A likely explanation for this finding is that the varus wedge resulting from a deeper medial heel skive (e.g. the 6 mm skive) has a greater surface area and intrudes further laterally within the heel cup. Therefore, in our study it is likely that the 6 mm heel skive encroached upon the lateral rearfoot mask, thereby increasing pressure within this mask. Therefore, it should be noted that although a deeper heel skive increases pressure under the medial heel it may also start to exert increased pressure under the lateral heel as it becomes more prominent in this region. The effect of this on rearfoot kinematics will largely depend on the resultant medial and lateral forces and how they combine with respect to the subtalar joint axis [8]. If these forces combine to result in an increase in the force medial to the subtalar joint axis and this increased force outweighs that of opposing forces (e.g. soft tissue forces), then a supination moment will still arise [24]. If, however, these forces result in an increase in the force lateral to the subtalar joint axis and this force outweighs that of opposing forces, then a pronation moment will arise.

With respect to the midfoot and forefoot, the effect on plantar pressures were not significantly different between each of the orthoses. This finding suggests that the 4 mm and 6 mm medial heel skives maintain the same effect on the midfoot and forefoot even though they increase pressure in the medial rearfoot. As a result, a medial heel skive is unlikely to provide either an undesirable or a favourable effect on midfoot and forefoot pressures. Accordingly, the medial heel skive, as determined in our sample of younger adult participants with a flat-arched or pronated foot type, does not have a significant effect on altering plantar pressure other than under the heel. Consequently, forefoot pathologies thought to benefit from a reduction in pressure, for example, would be unlikely to gain any added improvement from a medial heel skive modification being added to an orthosis.

The findings of this study support that clinicians should consider how an increase in medial rearfoot pressure may affect certain musculoskeletal conditions [8], particularly when using the 4 mm and 6 mm medial heel skive. When the medial heel skive modification was first described by Kirby he stated that it was contraindicated for conditions such as plantar heel pain, heel pad atrophy and calcaneal neuritis due to the heel cup contour [8]. That is, the varus wedge created under the heel by the skive could result in pathological increases in force to a heel that was already experiencing mechanically-derived pathology. Furthermore, as calcaneal spurs have been proposed to develop as an adaptive response to vertical compression [25-27], the long-term effects that any intervention, like the medial heel skive that increases such forces, may require further investigation.

The findings of this study need to be viewed in consideration of several limitations. First, the medial heel skive was initially proposed to increase the force exerted medial to the subtalar joint axis [8]. However, even though we evaluated the effects of the medial heel skive in our study, we did not use the subtalar joint axis location as an inclusion criterion. Instead, we chose to include participants based on their NNHT and FPI-6 (i.e. all participants had low-arched or pronated feet) as these measurements have previously been found to be both reliable and valid [9,11]. In addition, we did not analyse our pressure data in masks medial and lateral to the subtalar joint axis. Instead, we analysed forces relative to the medial and lateral halves of the weightbearing heel and midfoot (with more complex masking in the forefoot). Although there are methods to determine the spatial orientation of the subtalar joint they are generally limited to techniques that are either expensive, invasive or not clinically viable [28-30]. A simple clinical method for determining the location of the subtalar joint axis has been described [24], but the reliability and validity of this technique are yet to be adequately established. Furthermore, the technique is based on the theory that the subtalar joint acts as a single stationary hinge, whereas it is likely to function about a multitude of axes and as a complex of interdependent rearfoot joints [31]. Therefore, we chose not to determine the location of the subtalar joint axis as described by Kirby [8] for both screening participants and analysis of pressure data.

Second, despite the pedar-X® system having been shown to be a reliable and valid in-shoe plantar pressure system it only measures forces acting vertical to the insole [16,17,32] and it is likely that the forces that a foot orthosis exerts against the foot are more complex in nature [33-35]. As the pressure-mapping insoles have to contour to an orthosis rather than lie horizontally within a shoe they only record resultant force. As such, the shear component of such forces is not recorded and inherent measurement errors are likely to occur [33-35]. In addition, there are further concerns about validity with regard to spatial resolution [36]. Urry and Wearing [37,38] have shown potentially large errors in the accuracy of measuring contact area with pressure measuring systems such as the one that we used in our study. Despite the limitations of using in-shoe pressure measuring systems, they are commonly used when evaluating the mechanical effects of foot orthoses [20,22,23] and they are considered the most appropriate method to examine forces acting at the foot-orthosis interface [33]. Further development of in-shoe systems, such as the pedar-X®, is clearly warranted to ensure the most accurate assessment of in-shoe foot orthoses.

Third, standardised footwear and sockettes were used in this study to minimise their potential influence on the results. It is uncertain how the results of the study would differ if the orthoses were tested in more supportive footwear, as footwear itself can influence plantar pressures [39]. Fourth, the subjects used in this study were healthy and relatively young (mean age 24.1 years) with a flat-arched or pronated foot posture. Although the medial heel skive modification is indicated for flat-arched or pronated feet it remains unclear how the plantar pressure changes provided by the medial heel skive may correlate with patient outcomes – clinical trials are required to establish this. Finally, we recognise that the association between increased medial rearfoot pressure and its effects on other biomechanical parameters (e.g. moments about the subtalar joint axis) is still largely theoretical, and such an unequivocal relationship has not been established using robust scientific methods. In consideration of the aforementioned limitations, it would be beneficial for future studies to investigate the effects of the medial heel skive on other biomechanical parameters (e.g. kinematics), clinical outcomes and in wider populations and clinical presentations.

## Conclusion

The findings of this study indicate that a medial heel skive of 4 mm or 6 mm increases pressure under the medial heel in asymptomatic individuals with a flat-arched or pronated foot posture. In contrast, a smaller medial heel skive of 2 mm produced no significant change in pressure for the same region. Plantar pressures at the midfoot and forefoot were not affected by a medial heel skive of 2, 4, or 6 mm. Therefore, it is recommended that a medial heel skive of 4 mm or 6 mm can be used when an increase in medial rearfoot pressure is desired in individuals with flat-arched or pronated feet. Although, our findings should be considered in light of the limitations of the measurement apparatus used in our study, and caution is necessary when using this modification for people with medial heel pathology.

## Competing interests

The authors declare that there are no known conflicts of interest related to this project that could have influenced this manuscript.

## Authors' contributions

All authors were fully involved in the preparation of the study procedures. CYZ, RCF, and MGB collected the plantar pressure data and all authors were involved in data analysis. DRB was responsible for the preparation of the manuscript with all other authors involved in its review prior to submission for publication. The material within has not been and will not be submitted for publication elsewhere. All authors read and approved the final manuscript.
